# A web-based simulation of a longitudinal clinic used in a 4-week ambulatory rotation: a cohort study

**DOI:** 10.1186/1472-6920-9-8

**Published:** 2009-02-02

**Authors:** Rene WG Wong, Heather A Lochnan

**Affiliations:** 1 Department of Medicine, Division of Endocrinology, University Health Network – Toronto General Hospital, 200 Elizabeth Street, 12-EN-211, Toronto, Canada; 2 Department of Medicine, Division of Endocrinology, The Ottawa Hospital, 1967 Riverside Drive, 4th floor, Ottawa, Canada

## Abstract

**Background:**

Residency training takes place primarily on inpatient wards. In the absence of a resident continuity clinic, internal medicine residents rely on block rotations to learn about continuity of care. Alternate methods to introduce continuity of care are needed.

**Methods:**

A web-based tool, Continuity of Care Online Simulations (COCOS), was designed for use in a one-month, postgraduate clinical rotation in endocrinology. It is an interactive tool that simulates the continuing care of any patient with a chronic endocrine disease. Twenty-three residents in internal medicine participated in a study to investigate the effects of using COCOS during a clinical rotation in endocrinology on pre-post knowledge test scores and self-assessment of confidence.

**Results:**

Compared to residents who did the rotation alone, residents who used COCOS during the rotation had significantly higher improvements in test scores (% increase in pre-post test scores +21.6 [standard deviation, SD, 8.0] vs. +5.9 [SD 6.8]; p < .001). Test score improvements were most pronounced for less commonly seen conditions. There were no significant differences in changes in confidence. Residents rated COCOS very highly, recommending its use as a standard part of the rotation and throughout residency.

**Conclusion:**

A stand-alone web-based tool can be incorporated into an existing clinical rotation to help residents learn about continuity of care. It has the most potential to teach residents about topics that are less commonly seen during a clinical rotation. The adaptable, web-based format allows the creation of cases for most chronic medical conditions.

## Background

A shift of patient care from the inpatient to outpatient setting has taken place. Ideally, postgraduate training programs should ensure the curriculum matches this balance, yet internal medicine training programs in Canada continue to be dominated by clinical rotations on inpatient wards. Many obstacles exist in postgraduate medical education that limit the establishment of the most optimal experiences. First, block-format ambulatory rotations are often relied upon as settings to introduce continuity of care issues. Traditionally, ambulatory care rotations have been four – to six-week blocks of five to seven half-day clinics per week. Assigning residents to continuity or longitudinal clinics allow them to see patients over time, presenting opportunities to learn how to provide effective continuity of care. However, organizing high quality continuity experiences is often difficult especially when resident training is based at multiple sites. Second, residents are required to complete rotations in most, if not all, medical subspecialties; consequently the time spent per rotation is limited to one or two-month blocks which provide superficial exposure to clinical problems, and offer little continuity of care [1]. Third, resident learning in the ambulatory setting is dictated largely by chance; the stimulus for patient-centered teaching and learning is determined by how patients are scheduled clinics rather than a standardized method. Faculty often ask residents to see "new patients" as opposed to patients booked for follow-up care, which may reduce the impetus to focus their learning about continuity of care. The types of medical problems that each resident encounters cannot be standardized.

Continuity of care is one of the expressed goals of an ambulatory care experience. Longitudinal or continuity experiences are characterized by at least one half-day per week of ambulatory care experience in the same location with either a single or multiple preceptors. The benefits of longitudinal clinics include providing opportunities to follow patients over time to develop ongoing patient-physician relationships and learn about the unique challenges of managing chronic disease [2,3]. Due to the complexity of organizing continuity experiences for large numbers of residents most internal medicine training programs in Canada rely on block rotations to provide ambulatory experiences to its residents. Residents miss out on opportunities to learn about clinical issues that are specific to seeing patients longitudinally (for example, knowing how to adjust treatment, manage adverse events etc.) Alternate methods to enhance exposure to continuity of care issues within the constraints of existing residency training programs are needed.

Web-based teaching allows universal accessibility of the content, consistently delivers the same message to every learner and gives control of learning to the student without distracting them from their expected clinical responsibilities [4-8]. In the context of residency training programs that do not offer continuity clinics, the role of web-based simulations continuity experiences has not been defined. Numerous published studies evaluating the use of web-based teaching at both the undergraduate and postgraduate levels have noted increased learner satisfaction as well as improvements in pre-post test scores. However we were not able to identify any studies of the effect of web-based simulations of continuity of care as an adjunct to a block-style clinical rotation at the postgraduate level. The most applicable study compared web-based to traditional learning formats within an internal medicine resident continuity clinic. Residents preferred the web-based learning modules but no difference in pre- to postintervention test scores were noted [7]. However, web-based learning was evaluated as an adjunct to a preexisting continuity experience, and the results may not be applicable to programs without this experience. Another pertinent study showed that the use of a 'stand-alone' online curriculum in ambulatory care was rated favorably by residents and program directors, and significantly improved test scores [9]. In this study the degree to which the curriculum content focused on medical issues that arise during continuing or follow-up care was not explicitly mentioned.

In the absence of published reports demonstrating the use of web-based technology to simulate what occurs in a continuity experience, we designed Continuity of Care Online Simulations (COCOS), an online learning tool that simulates the longitudinal care of patients with chronic diseases. We report our experience in developing this web-based learning tool and its use by internal medicine residents whose only ambulatory experiences consist of short 4 to 8 week blocks rather than continuity or longitudinal experiences. We determined its effects (as an adjunct to a 4-week ambulatory rotation) on resident knowledge and confidence in providing continuing care of patients in the ambulatory setting.

## Methods

### Development of continuity of care objectives

An ambulatory-care curriculum emphasizing continuity of care was designed using a 6-step approach to curriculum development [10] and validated by a panel of two medical educators, both endocrinologists. "Continuity of care objectives" were categorized under 5 headings (Table 1) that include topics pertinent to seeing a patient for the first time (e.g. "Diagnostic tests", "differential diagnosis") as well as topics specifically relevant to follow-up or continuing care (e.g. "monitoring treatment efficacy", "managing adverse effects of treatment", "adjusting treatment"). For each objective, disease-specific objectives were written for the following endocrine conditions: 1) type 2 diabetes, 2) type 1 diabetes, 3) hyperthyroidism, 4) thyroid nodules, 5) hyperprolactinemia. When selecting these conditions, it was our intent to select both commonly and uncommonly seen conditions to examine this would have an impact on the effects of computer simulations. Prior to the study, we had obtained resident reports of their exposure to various endocrine conditions as part of their evaluation of the clinical rotation. This was the basis for their selection; type 1 and 2 diabetes were very commonly seen, hyperthyroidism and thyroid nodules were seen at a moderate frequency, and hyperprolactinemia was seen uncommonly.

**Table 1:** Ambulatory care objectives used to structure COCOS

Diagnosis
1. Identify pertinent features on history and physical examination
2. Describe diagnostic tests used to evaluate a patient, including their cost-effectiveness
3. List the differential diagnosis for a patient based on his/her clinical presentation

Natural history

1. Classify a patient's current state in the natural history of a chronic condition
2. Describe, to a patient, the expected course of their condition without treatment
3. Order tests, if necessary, to monitor the course of a chronic condition

Treatment

1. Describe, to a patient, and compare treatment options including: mechanism of action; benefits, including efficacy and time course; risks, including adverse effects
2. Order tests, if necessary, to monitor treatment efficacy and/or adverse effects
3. Analyze data to determine changes in treatment that is required
4. Discuss an appropriate duration of treatment for a chronic condition
5. Detect if treatment has failed, and select alternate treatment options

Special situations

1. Provide prepregnancy counseling to a woman with a chronic condition, including: how her condition will affect fertility; how or if her condition will change during pregnancy; how the condition will affect her during pregnancy; how the condition will affect the fetus during pregnancy; the likelihood her baby will develop the same condition, management or treatment that should take place prior to conception
2. Formulate a management plan for a pregnant woman with a chronic medical condition
3. Manage a patient with a chronic medical problem during the peripartum period
4. Formulate a management plan for a woman with a chronic medical condition during the immediate and later post-partum period

Practice management

1. Discuss the criteria by which requests for consultation should be prioritized as urgent
2. Discuss an appropriate time frame to arrange follow-up care in the case of: a patient in whom the diagnosis has not been made; a patient in whom you've initiated therapy; a patient on therapy who has not been stabilized; a patient on therapy who has been stabilized
3. Discuss factors that influence whether a patient can be followed his/her primary care physician for a medical condition
4. Describe key recommendations that should be made to a primary care physician who will be following a patient for a medical condition

Using the generic objectives as its foundation, we created a storyboard depicting sequential clinical appointments for a patient with a chronic medical condition. The storyboard was used as a template for all cases to ensure that each of the objectives (table 1) was discussed. Case authors were only required to add disease-specific information to the generic template to create a case. Case studies were created for: type 1 diabetes (commonly seen), Graves disease (moderately seen), hyperprolactinemia (uncommonly seen). These topics were specifically selected based on the frequency in which past residents reported seeing patients with those conditions during a typical 4-week endocrinology rotation. Each case averaged 7500 words and was reviewed by at least two endocrinologists to establish content validity.

A budget of $5000 Canadian was used to create the website for use in the pilot study. Only an Internet browser was required to utilize the website which was accessible using pre-assigned usernames and passwords. Each page of a case depicts a simulated appointment with a patient and the resident is asked to make one or more clinical decisions and/or answer questions posed by the simulated patient. Immediate feedback is shown in a pop-up message window or on the subsequent page depicting the subsequent clinic appointment. Residents could complete self-assessment quizzes before and after the case study.

### Protocol

**Figure 1:**
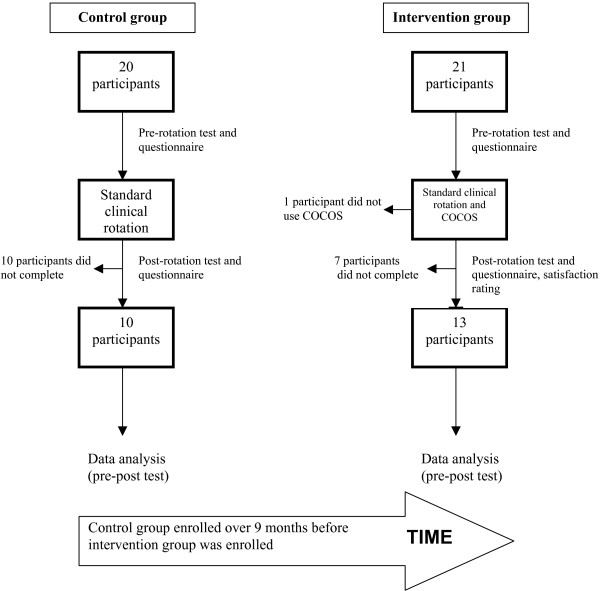
**Flow diagram showing the structure of the cohort study**.

At the University of Ottawa Medical school (Ottawa, Canada), medical residents assigned to the endocrinology rotation were invited to participate. Attending physicians were aware of the objectives for the rotation, but were not made aware of the specific objectives for COCOS. We designed this study as a single institution, double cohort trial to investigate whether the use of COCOS during a clinical rotation can significantly improve learning and confidence of medical residents in the longitudinal management of patients with endocrine disorders. The hospital's ethics board approved this study. Written consent was obtained from all study participants. Figure 1 shows an algorithm of the control and intervention group analysis. The control group consisted of residents completing the endocrinology rotation. They were provided specific reading material and printed guidelines on endocrine topics. The intervention group completed the same rotation, but was expected to complete COCOS before the end of the rotation. Thus this group was using COCOS as an adjunct to the existing rotation. No designated time was allocated to use COCOS, which was accessible using unique usernames and passwords. The control group was recruited and assessed over a 9-month period before the intervention group to ensure users of COCOS did not discuss or share the website content with members of the control group.

### Outcome measures

#### A. Pre-Post test

Based on our ambulatory care curriculum, we developed short answer questions addressing the objectives for each disease. Questions were in the context of patient scenarios viewed from the perspective of a general internist. A panel of medical educators established content validity of the tests. Construct validity was established by the administration of the test to full-time division members in endocrinology. The final version of the pre- and post-tests contained an equal distribution of questions pertaining to topics specific to seeing a patient during an initial consultation, and topics relevant to seeing a patient in follow-up or during continuing care. Questions were also equally distributed by condition (type 1 diabetes, type 2 diabetes, hyperthyroidism, hyperprolactinemia, polycystic ovarian syndrome). To control for different levels of baseline knowledge among residents, we measured the difference between pre-rotation and post-rotation scores as the outcome variable.

#### B. Self-rating of confidence

Residents self-rated their confidence in managing patients with endocrine and other medical conditions on a five-point Likert scale. Questions were subdivided into ratings for two types of patient encounters: "initial consultation" and "follow-up appointment". To control for the different level of baseline confidence among residents, we measured the difference between pre-rotation and post-rotation scores as the outcome variable.

#### C. Rotation questionnaire

Residents were asked to indicate how often they saw a patient with each condition during their 4-week rotation. This allowed us to control for the amount and type of clinical content encountered during the rotation when analyzing the results of our outcomes, and allowed us to determine if there was a differential effect of COCOS depending on the type and commonality of a clinical topic. We did not review clinic records to measure the exact numbers of patients seen by each resident. Members of the intervention group were also asked to rate their level of agreement with statements about COCOS on a 5-point Likert scale (1 = strongly disagree, 5 = strongly agree) with respect to its contribution to their learning.

### Statistical analysis

P values were calculated between pre and posttest results of the matched test results within groups using Students' t test (paired analysis), and between groups using a Student's t test (unpaired analysis). Median rankings of self-confidence pre- and post-rotation were calculated; to test for differences in the median values between the start and end of rotation, and between groups, a non-parametric one-way analysis of variance statistic was used. Survey Likert scores were analyzed using the Wilcoxon signed-ranked test with the median of the scale (three) as a reference. All analyses were performed using a two-sided alpha level of .05. To detect an improvement of knowledge score of 20 percentage points with an estimated standard deviation of 20 points, a 2-tailed alpha error of 0.05 and a power of 0.80, sample size was calculated at a minimum of 16 resident participants.

## Results

From July 2003 to June 2005 a total of 45 residents participated in our clinical rotation. Four residents were excluded because they were absent for the pre-rotation test. Of the 20 residents enrolled into the control group, 10 did not complete the post-rotation test and questionnaire. Of the 21 residents enrolled for the intervention group, 7 did not complete the post-rotation test and questionnaire and 1 resident did not use COCOS during the rotation. 10/20 (50%) residents in the control group and 13 of 21 (62%) residents in the intervention group were included for evaluation. Characteristics of the participating residents are shown in Table 2.

**Table 2:** Characteristics of participating residents

	Control (n = 10)	Intervention (n = 13)
Average age (years)	28.8	29.5

Gender:		

Male	5 (50%)	6 (46%)
Female	5 (50%)	7 (54%)

Previous degrees:		

Bachelor of Science	10	12
Bachelor of Education	0	1
Master's degree	1 *	2 *

Level of training:		

2^nd^-year residents	5 (50%)	9 (69%)
3^rd^-year residents	5 (50%)	4 (31%)

Prior clinical rotations in endocrinology		

0 months	6 (60%)	9 (69%)
1 months	4 (40%)	3 (23%)
2 months or more	0 (0%)	1 (8%)

Career goal		

General internal medicine	1 (10%)	1 (8%)
Endocrinology	0 (0%)	0 (0%)
Other medical subspecialty	7 (70%)	8 (62%)
Undecided	2 (20%)	4 (30%)

Opinions regarding need for emphasis on continuity of care in residency:		

More emphasis required	7 (70%)	7 (54%)
No change required	3 (30%)	6 (46%)
Less emphasis required	0 (0%)	0 (0%)

Self-reported use of internet		

Full knowledge of internet use	10 (100%)	13 (100%)
Daily use of internet	10 (100%)	13 (100%)

Median estimates of the number of patients seen during the endocrine rotation:		

Type 2 diabetes	> 10	> 10
Type 1 diabetes	5–10	5–10
Hyperthyroidism	5–10	3–5
Thyroid nodules and/or goiter	5–10	3–5
Hyperlipidemia	5–10	5–10
Hyperprolactinemia	1–2	1–2
Polycystic ovarian syndrome	1–2	1–2
Adrenal insufficiency	1–2	1–2
Pituitary disorders	1–2	1–2
Hyperparathyroidism	0	0
Osteoporosis	0	1–2

* Some participants had more than one degree at the time of the study

### Knowledge improvements

Cronbach's alpha for the test questions was .753 (pre-test) and .851 (post-test) suggesting acceptable reliability. There were no significant differences between the two groups' pre-rotation test scores. There was no difference in pre-rotation test scores between second and third year residents but residents who had completed one or more endocrinology rotations in the past (n = 8) had slightly higher pre-rotation scores (mean score 64.8% vs. 59.2%).

Table 3 shows test scores for both groups. The pre-test and post-test scores were 62.2% and 68.2% respectively for the control group. Significant increases were noted for questions pertaining to seeing patients during an initial consultation (p = .023) but not for questions pertaining to seeing patients in follow-up (p = .717). Significant increases were noted for questions pertaining to the management of type 1 diabetes (p = .041) and type 2 diabetes (p = .019), which were noted by residents to be the most common conditions seen during the rotation. The pre-test and post-test scores were 60.9% and 82.6% respectively for the intervention group. Significant increases were noted for questions pertaining to seeing patients during initial consultation (p < .001) and during follow-up visits (p < .001), and for all diseases other than thyroid nodules for which the results approached but did not attain statistical significance (p = 0.06).

**Table 3:** Mean total scores for pre-rotation and post-rotation tests (scores are expressed as a total score out of 100)

	Control group			Intervention group		
	Pre-rotation	Post-rotation	P-value	Pre-rotation	Post-rotation	P-value

Total score	62.2	68.2	.023 *	60.9	82.6	< .001*

By type of patient encounter:						

Initial consultation	59.8	70.1	.023*	58.6	81.6	< .001*
Follow-up care	65.2	66.3	.717	63.6	83.6	< .001*
Diagnosis	53.2	67.1	.015*	50.7	83.6	< .001*
Initiating therapy	64.9	72.5	.072	64.4	80.1	.001*
Natural history	52.2	71.2	.094	49.6	87.0	< .001*
Managing treatment	60.4	52.3	.116	54.9	74.9	.003*
Pregnancy	63.4	63.8	.944	63.5	85.5	.001*
Practice management	73.6	82.8	.072	71.2	80.4	.039*

All COCOS cases†:	66.5	70.4	.152	61.5	80.1	< .001*

Type 1 diabetes	70.7	79.5	.041*	68.2	85.1	< .001*
Graves disease	68.8	65.3	.537	59.6	76.6	.018*
Hyperprolactinemia	54.7	59.3	.554	50.6	77.2	.001*

All non-COCOS cases††	60.3	65.1	.285	60.3	84.9	<.001*

Type 2 diabetes	58.0	76.3	.019*	61.9	90.5	<.001*
Thyroid nodules	68.2	56.4	.008*	68.6	78.3	.060
Polycystic ovarian syndrome	48.8	56.1	.434	52.4	81.7	<.001*

* Significant p-value (< 0.05), paired t-test

† : cumulative score for questions pertaining to topics for which COCOS cases were written (type 1 Diabetes, Graves disease and Hyperprolactinemia)

††: cumulative score for questions pertaining to topics for which COCOS cases were not written (type 2 Diabetes, thyroid nodules, polycystic ovarian syndrome)

**Figure 2:**
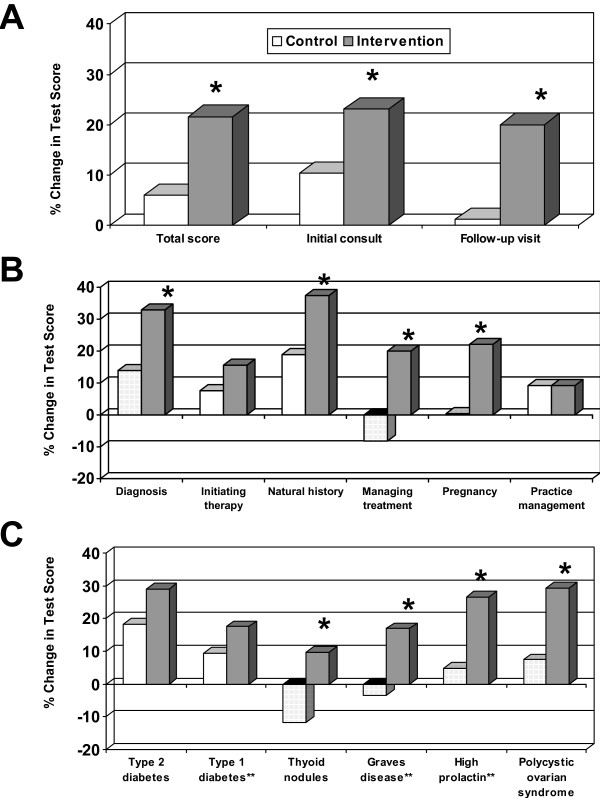
**Changes in test scores between the start and end of a four-week rotation in endocrinology**. White bars indicate control group, Grey bars indicate intervention group. A. Total score and by type of patient encounter. B. Scores on generic topics. C. Scores by disease type. *p < 0.05 for comparison of changes in test scores between the control and intervention group. ** denotes a condition for which a COCOS case was written.

Figure 2 shows the change in test scores from the start to the end of the rotation. Compared to the control group, the intervention group had significantly greater improvements in total test scores, as well as scores for questions pertaining to seeing patients during initial consultation and follow-up appointments. The most notable improvements were noted for "diagnosis", "managing ongoing therapy" and "management during pregnancy". There was no significant difference in "practice management". The intervention group had significantly greater improvements in test scores for questions pertaining to Graves disease and hyperprolactemia (conditions for which COCOS cases were written, and that were noted to be uncommonly seen during the rotation) and thyroid nodules (for which a COCOS case was not written). There were no significant differences for type 1 diabetes (+8.8 vs. + 16.9% change, p = .10), a commonly seen condition, despite the availability of a COCOS case for that topic. The intervention group had a greater improvement in test scores for questions regarding type 2 diabetes, the most common condition seen during the rotation, but this did not reach statistical significance (control +18.3 v intervention +28.6% change, p = .19).

### Assessment of confidence

Table 4 shows residents self-ratings of confidence (before and after the rotation) to manage patients as part of an "initial consultation" and during a "follow-up appointment". By the end of the rotation, residents in both groups reported being most confident managing patients with diabetes and least confident managing hyperprolactinemia and polycystic ovarian syndrome. Improvements in the control group's self-ratings of confidence between the start and end of the rotation, rated on a five-point Likert scale, were significant for Graves disease (2.75 pre-rotation to 3.45 post-rotation, p < 0.01), thyroid nodules (2.42 to 3.45, p < .01), hyperprolactinemia (2.08 to 2.95, p = .04) and polycystic ovarian syndrome (2.08 to 2.72, p = 0.04). In the intervention group, improvements were similarly noted for Graves disease (2.69 to 3.54, p < .01), thyroid nodules (2.54 to 3.61, p < .01), hyperprolactinemia (1.85 to 3.31, p < .01) and polycystic ovarian syndrome (2.15 to 2.92, p = .01). There were no differences between groups at the start or end of rotation regardless of disease or type of encounter (initial consultation vs. follow-up visit).

**Table 4:** Mean self-ratings of residents' confidence to manage different conditions (scores are expressed on a 5-pt Likert scale, 1 = no confidence, 5 = extremely confident).

	Control (initial consultation)			Intervention (initial consultation)		
Condition	Pre-rotation	Post-rotation	P-value	Pre-rotation	Post-rotation	P-value

Type 1 diabetes	3.92	3.72	.845	3.69	4.08	.206
Graves disease	2.75	3.45	.005*	2.69	3.54	.002*
Hyperprolactinemia	2.08	2.95	.035*	1.85	3.31	< .001*
Type 2 diabetes	4.17	4.00	.854	3.92	4.31	.136
Thyroid nodules	2.42	3.45	.001*	2.54	3.61	< .001*
Polycystic ovarian syndrome	2.08	2.72	.044*	2.15	2.92	.007*

P values are for comparisons between pre- and post-rotation ratings. * denotes significant p-value (< 0.05).

### Resident satisfaction

Eleven of 13 residents in the intervention group completed the COCOS survey. The response was overall very positive, with 91% of residents recommending its use as a standard component of the endocrine rotation and 100% of residents recommend the use of COCOS in other rotations. Features of COCOS that were rated highest were the provision of immediate feedback and the emphasis on continuity of care. Features rated lowest were the length of each case and amount of repetition within the case content. Each case took between 15–20 minutes to complete, and 10 of 11 residents felt the ideal number of COCOS cases to complete in a one-month rotation was between 4–6. Residents reported using COCOS at home as well as within the hospital. Residents' levels of agreement with statements about COCOS are shown in Table 5.

**Table 5:** Residents' level of agreement regarding the COCOS learning modules (1 = strongly disagree, 5 = strongly agree; * Wilcoxon Signed-rank test)

Statement	Mean Rating ± SD (p value)
The preparation material and instruction for using COCOS was adequate.	4.45 ± .52 (< .001)
The time available during this rotation was adequate to allow use of COCOS on my own time.	4.54 ± .52 (< .001)
COCOS allowed me to learn about continuity of care I would not otherwise have learned.	4.45 ± .69 (< .001)
COCOS provided me with more exposure to endocrinology I would not otherwise have had.	4.27 ± .79 (.003)
COCOS enhanced my self-directed learning.	4.54 ± .69 (< .001)
After this rotation, I plan to use the COCOS cases in endocrinology as a resource.	4.09 ± 1.04 (.010)
COCOS cases in more endocrinology topics would benefit residents' learning during this rotation.	4.54 ± .69 (< .001)
COCOS cases in other medical topics should be used on other rotations.	4.54 ± .52 (< .001)
COCOS increased my interest in endocrinology.	4.09 ± .94 (0.01)
COCOS should be included as a standard component of the endocrinology rotation.	4.36 ± .67 (.003)

## Discussion

We have described the successful implementation of an online resource used by residents in a one-month ambulatory experience that simulates the clinical decisions that needed to be made when seeing patients in a longitudinal clinic. Our use of simulations allowed the residents to practice patient care in a safe environment and provided immediate feedback on their patient-care decisions. The content and format was rated highly with the majority recommending its ongoing use not only in our rotation but also in others during their residency. Residents accessed COCOS on their own time without the need for direct involvement of faculty. It was not perceived to be labor or time intensive for the residents; each case required approximately 20 minutes to complete, consistent with other reports [9]. Completing four to six cases in a four-week period appears to be an acceptable expectation of residents. Even though we applied the model to topics in endocrinology, its generic template facilitates writing cases in other medical subspecialties with a strong ambulatory component.

COCOS is unique in its emphasis on the competencies required to provide follow-up or continuing care to patients. To our knowledge this is the first study that has investigated the effect of web-based simulations of continuity of care as an adjunct to a clinical rotation at the postgraduate level. "Stand-alone" internet curricula in ambulatory have been rated highly by internal medicine residents and resulted in improved test scores [9,11] but lack of control groups in these studies did not allow comparisons to 'traditional' teaching formats. Our use of a control group allowed us to make a comparison with our 'traditional' clinical rotation to help us determine whether it is useful as an adjunct rather than a replacement. Other interventions used at the postgraduate level in internal medicine ambulatory settings did not provide as much emphasis on continuity of care [12].

We did not measure the number of resident encounters with patients for each category (initial consult or follow-up appointment), but traditionally residents are asked to see new patients (i.e. initial consult) rather than patients returning to clinic. Students' learning in the clinical setting is more often opportunistic and thus more learning is likely to occur around topics that are common compared those that are less commonly seen. Even though the use of COCOS resulted in improved test scores for topics relevant to the "initial consult", a more pronounced improvement was for topics relevant to seeing patients during the "follow-up visit". Thus COCOS may be most beneficial to improve learning around types of patient encounters (i.e. follow-up appointments) that are less frequent during a short clinical rotation. Similarly, COCOS was more beneficial for diseases or conditions less commonly seen during the rotation. The use of COCOS cases for Graves disease and hyperprolactinemia resulted in a significant improvement in test scores, in contrast to a non-statistically significant improvement for questions pertaining to type 1 diabetes. Comments left by the residents included suggestions to create cases for other endocrine disorders uncommonly seen such as adrenal insufficiency and primary hyperparathyroidism. Thus the use of COCOS as an adjunct to a clinical rotation should emphasize more "follow-up" or "continuing care" for conditions like diabetes that are commonly seen, but include content for both "initial consult" and "follow-up" issues for uncommon conditions.

It is noteworthy that the use of COCOS resulted in greater test score improvements in topics for which there were no COCOS cases (thyroid nodules, PCOS). There are two possible explanations for this observation. Residents in the intervention group may have had better exam-taking abilities compared to those in the control group. Alternatively the use of COCOS results in qualitative changes in the learning process that may result in more efficient or improved learning, as has been noted in some studies outside of medical education [13,14]. 91% of residents reported COCOS enhanced self-directed learning and our group of 6 full-time faculty members anecdotally reported residents asked more questions about follow-up care, lending support to this hypothesis. However for this to occur COCOS would have to be used early in the rotation and we did not determine when in the rotation it was used by each resident.

Our residents' confidence in managing different types of endocrine conditions is similar to what is reported in the literature [15]. In contrast to the difference in knowledge gains with the use of COCOS, there were no significant differences between groups in the changes in confidence. Residents may overrate themselves at the start of the rotation or underrate themselves at the end of the rotation when compared with ratings of their supervisors [16,17]. The latter may be especially true for residents who used COCOS if they perceived they had done poorly during the case simulations. A retrospective self-analysis of how residents thought their confidence changed over the course of the rotation would be insightful.

The use of COCOS during the rotation proved to be advantageous compared to the rotation alone that included the provision of printed material. One of the key reasons for conducting this study relates to how there is a lack of reading material that adequately covers many of the "continuity of care" learning objectives. The reading material and printed guidelines provided to the control group were primarily review articles that only briefly address the COCOS learning objectives. We did not provide the control group with a paper-based version of the COCOS content. We feel that COCOS has many features that provide advantages over a stand-alone, paper-based source of information. Simulated cases provide residents with opportunities to build on knowledge gained from their clinical experience thus promoting a deeper learning. While we did not include multimedia content for the purpose of this pilot study, images, sounds and video clips can be easily incorporated into any case to add realism to the simulations. The option to complete online pre- and post-case self-assessment quizzes captures their attention and capitalizes on the learner's motivation [18]. The interactive format including the ability to record resident answers to questions, was adopted not only to better engage the learners [19], but also to assist course administrators identify gaps in knowledge and in the curriculum as part of a needs analysis.

Manually authoring unique case scenarios with potentially different sequences, content and variables, is an extremely labor-intensive undertaking that limits their availability or reproducibility [20]. By using a generic template design for COCOS, disease-specific information was easily added to allow the creation of cases. In COCOS residents are asked to answer questions or make clinical decisions, and regardless of the type of response, the storyline continues in its preset course. A switch to a more probabilistic type of simulation where an action could result in a number of theoretic outcomes would improve the level of realism.

The text-based format of the cases allows for easy modification in light of changes in clinical evidence or to adjust the case sequence for disease in which certain topics may not be applicable. For example, the page devoted to "Medical management during pregnancy" could be omitted or interchanged with other special situations such as "Preparation of surgery" with relative ease. Opportunity exists for a wide application of COCOS beyond the postgraduate level, including both undergraduate and continuing education.

This study has several limitations. It was conducted at a single training program and the structure and administration of our rotation may not be representative. We only focused on "continuity of care" from the perspective of the CanMEDS role as a Medical Expert. There are elements of longitudinal clinical practice that are difficult to simulate (e.g. reviewing the chart, refreshing oneself on the patient's problems, retrieving missing information, understanding patient-doctor relationships, family dynamics and community resources) [3] that require competencies in other CanMEDS roles such as Manager, Communicator and Professional. Only hands-on experience may be adequate to teach our trainees about these elements. We did not randomize residents within each rotation to avoid contamination. We are somewhat reassured that the structure and administration of the rotation did not differ between the two groups, and the self-reports of the numbers of types of patients seen by the two groups were similar. Even though residents may have been enrolled at different times, there was no difference in baseline test or confidence scores. 50% of the control group and 31% of the intervention group were in their third-year of residency. If clinical experience influenced outcomes, one would anticipate that the group with more experience would perform better on the knowledge assessment. Alternatively, more senior residents may have better-established learning habits and thus may be less likely to benefit from an online intervention compared to more impressionable junior residents. Regardless, despite being at a relatively earlier stage of training, the intervention group had greater improvements in test scores; if randomization resulted in an equal distribution by postgraduate year, the differences between groups may have been more pronounced. Similarly, if participants were randomized and contamination had occurred, the control group's test scores could be artificially raised, and thus the results would still be significant if the intervention group demonstrated greater improvements in knowledge. Alternating the control with the intervention group from month to month is a potential way to improve validity and avoid contamination.

When interpreting the results, it is not known whether the improvements in test scores seen in the intervention group can be attributed to a computer-dependent feature in COCOS. Future studies comparing paper-based to a web-based presentation of COCOS content, or comparing two different versions of COCOS (to measure the impact of change in a single feature while holding others constant) would be useful. The net worth of COCOS on the learning gain, relative to the resource costs to develop and maintain it, remains unknown. We did not measure the amount of time residents spent completing the online modules nor did we survey residents on whether they felt the use of COCOS resulted in a more efficient use of time to learn about its specific topics, thereby allowing them to allocate the saved time for learning in other curriculum areas. Thus it is theoretically possible that the time allocated to the use of COCOS could have taken away from other learning opportunities. We also did not measure whether the implementation of COCOS resulted in staff physicians being able to save time and/or observe improvements in their other academic responsibilities. While resident opinions may not be the best measure of worth, the overwhelming response of residents who used COCOS was very positive and did allow them to better learn about topics that they would not have otherwise been able to do.

Forty-four percent of our total participants did not complete the post-test, and their pre-test scores were not included in analysis. We did not systematically measure reasons for non-completion. It was explicit that participation in the study was voluntary and we suspect that these residents gave less priority to completing the post-test in favor of other aspects of the rotation. This could have biased the results, in two ways. First, if these residents had a lower baseline level of knowledge, our actual results would have ultimately underestimated the changes in pre-post test scores. In contrast, if these residents had higher pre-test scores, our results would have overestimated the changes. However, when analyzing the pre-test scores of who did not complete the study, there was no difference between groups and we feel the dropout rate did not influence our interpretation of the data. Second, not completing the post-test could be an indicator of relatively weaker skills or level of interest in self-directed learning and thus, our final results would not be applicable to them. It is unclear as to why one resident assigned to the intervention group did not end up using the COCOS program, but there were no reports however of technical difficulties with accessing the website.

The objective was to determine the effect of COCOS as an adjunct learning tool within an existing clinical rotation, and we can not decipher whether the technology itself or the content (using technology as its vehicle for presentation) contributed most to the benefits. Specific studies are needed to determine whether different types of such presentation elements that we included (e.g. popup windows, interactivity) provide enough learning benefit to justify their higher costs. A comparison of the effects of case-based content and non-case-based content would be an informative comparison of two different instructional methods to present the topics in our curriculum. It would also be interesting to compare the effects of the use of COCOS in a short, block rotation to the longitudinal clinical experience. We are currently studying whether the knowledge gained from using COCOS leads to long term retention and improvements in clinical performance.

## Conclusion

In summary, we have described and evaluated an innovative way of teaching continuity care objectives using a web-based module in endocrinology in which cases are presented through several episodes across virtual time. As an adjunct, not replacement, to clinical experiences it can significantly improve residents' learning of ambulatory care endocrinology. Cases can be written for any chronic medical condition using a generic template based on preset objectives. The most pronounced benefits are seen for topics that are uncommonly seen during their clinical rotation or issues that arise in follow-up care. Our results have significant relevance to postgraduate medical training, where traditionally short, block-style rotations limit exposure to the wide range of patient problems and logistic difficulties and demands for increasing faculty productivity challenge traditional teaching efforts. Interested parties may sample the website by directing their browser to  and use the guest username : *endocrino *and password : *cocos *.

## Competing interests

The authors declare that they have no competing interests.

## Authors' contributions

RW conceived of the study, analyzed and interpreted the data, and drafted the manuscript. HL participated in the design of the study and coordination and helped to draft the manuscript. All authors read and approved the final manuscript.

## Pre-publication history

The pre-publication history for this paper can be accessed here:


